# Gene order data from a model amphibian (*Ambystoma*): new perspectives on vertebrate genome structure and evolution

**DOI:** 10.1186/1471-2164-7-219

**Published:** 2006-08-29

**Authors:** Jeramiah J Smith, S Randal Voss

**Affiliations:** 1 Department of Biology and Spinal Cord and Brain Injury Research Center, University of Kentucky, Lexington, KY, 40506, USA

## Abstract

**Background:**

Because amphibians arise from a branch of the vertebrate evolutionary tree that is juxtaposed between fishes and amniotes, they provide important comparative perspective for reconstructing character changes that have occurred during vertebrate evolution. Here, we report the first comparative study of vertebrate genome structure that includes a representative amphibian. We used 491 transcribed sequences from a salamander (*Ambystoma*) genetic map and whole genome assemblies for human, mouse, rat, dog, chicken, zebrafish, and the freshwater pufferfish *Tetraodon nigroviridis *to compare gene orders and rearrangement rates.

**Results:**

*Ambystoma *has experienced a rate of genome rearrangement that is substantially lower than mammalian species but similar to that of chicken and fish. Overall, we found greater conservation of genome structure between *Ambystoma *and tetrapod vertebrates, nevertheless, 57% of *Ambystoma*-fish orthologs are found in conserved syntenies of four or more genes. Comparisons between *Ambystoma *and amniotes reveal extensive conservation of segmental homology for 57% of the presumptive *Ambystoma*-amniote orthologs.

**Conclusion:**

Our analyses suggest relatively constant interchromosomal rearrangement rates from the euteleost ancestor to the origin of mammals and illustrate the utility of amphibian mapping data in establishing ancestral amniote and tetrapod gene orders. Comparisons between *Ambystoma *and amniotes reveal some of the key events that have structured the human genome since diversification of the ancestral amniote lineage.

## Background

**Figure 1:**
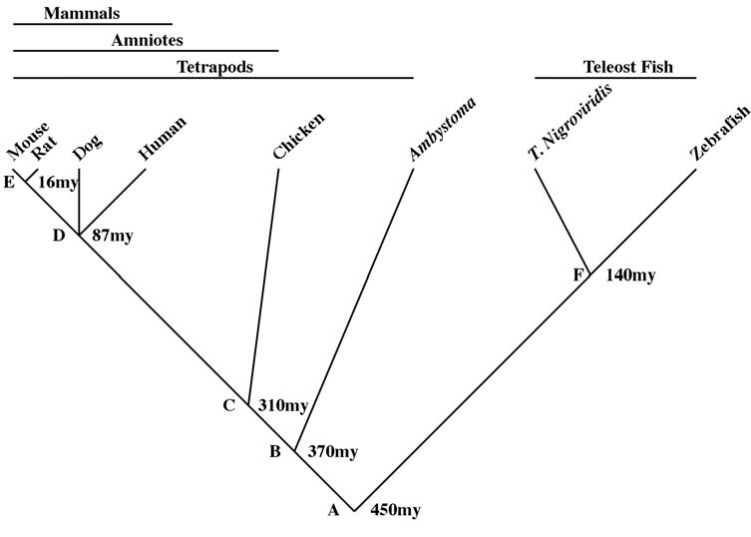
An abridged phylogeny of the vertebrates showing the species used and divergence times. Letters mark ancestral nodes: A – the euteleost (bony vertebrate) ancestor [62], B – the tetrapod ancestor [63], C – the amniote ancestor [64, 65], D – the (eutherian) mammalian ancestor [66], E – the (murid) rodent ancestor [66], and the teleost fish (euteleostei) ancestor [67, 68].

Amphibians (salamanders, frogs, and cecilians) arise from a branch of the vertebrate evolutionary tree that is juxtaposed between aquatic fishes and more terrestrial amniotes (Figure 1). This phylogenetic location therefore positions amphibians to provide important comparative perspective for reconstructing character changes that have occurred during vertebrate evolution. For example, the amphibian perspective is essential for understanding molecular, developmental, and morphological changes of appendages that are associated with the transformation of obligatorily aquatic fish to terrestrial tetrapods [1-4]. In addition, because amphibians are the most basal tetrapod lineage, the amphibian perspective is essential for understanding the evolution of amniote characteristics among the "higher vertebrate" groups. Although many studies have exploited the phylogenetic position of amphibians for comparative perspective, very few comparisons have been made at the genome level. Here we present results from the first broad-scale comparison of genome structure between an amphibian and other representative vertebrate taxa.

One of the most fundamental structural characteristics of genomes is the order in which protein-coding genes are arranged on chromosomes. Gene order is determined using one of several approaches, including physical mapping, linkage mapping, and whole genome sequencing. The most powerful approach is whole genome sequencing [5-9], but only if the final product is a complete (or nearly complete) genome assembly. Physical mapping refers to the direct localization of a gene to a whole or partial chromosome, for example by the method of somatic cell hybridization [10-13] or chromosome in-situ hybridization [14-17]. In comparison to these physical genome approaches, genetic linkage mapping refers to the approach of estimating recombination frequencies among loci (genes) in a segregating cross for the purpose of ordering genes into linkage groups [e.g. [18]]. Ultimately, the genomic approach taken to order genes in a particular species is determined by genome characteristics and the availability of resources. For example, the extremely large genome size of some amphibians makes it difficult to justify a whole genome sequencing effort at this time [19,20]. However, genetic linkage mapping is an efficient strategy for amphibians because large numbers of offspring can be obtained from segregating crosses, thus allowing accurate estimates of map position [21].

Until recently, there were few amphibian gene order data available for comparative analyses of vertebrate genome structure [22,23]. Much physical genome sequence has been collected recently for an anuran amphibian (*Xenopus tropicalis*), but this sequence has not yielded a complete genome assembly and there are no large-scale genetic maps for *Xenopus *that can be used in comparative studies [24]. The recently developed genetic linkage map for the salamander genus *Ambystoma*, however, now provides an amphibian resource that can provide structural and evolutionary perspective at the genomic level [21]. Here we report on the largest gene order dataset ever obtained for an amphibian. We use this dataset to describe the extent to which gene orders have been conserved between *Ambystoma *and other representative vertebrate species with assembled physical genome maps. We also describe several examples that demonstrate the importance of the amphibian genome perspective for reconstructing gene orders of the ancestral tetrapod and amniote genomes, and for understanding the importance of gene order rearrangement in vertebrate evolution.

## Results

### Identification of putative orthologs

We searched 491 protein-coding marker sequences from the *Ambystoma *genetic map against the genome sequences of human (*Homo sapiens*), mouse (*Mus musculus*), rat (*Rattus norvegicus*), dog (*Canis familiaris*), chicken (*Gallus gallus*), zebrafish (*Danio rerio*), and freshwater pufferfish (*Tetraodon nigroviridis*) to identify presumptive orthologs. For each search, we defined orthologs as the BLAT hit with the highest bitscore, plus all other hits within 1% of the highest bitscore. Using this definition, orthologs for 344 (70%) *Ambystoma *sequences were identified within the genome of at least one species in the reference set. Alignment summaries for all presumptive *Ambystoma*-vertebrate orthologs are provided as supplementary data [see Additional file 1]. The number of *Ambystoma *orthologs varied among species, ranging from 237 – 322. A low proportion of *Ambystoma *orthologs aligned to more than one presumptive ortholog in all comparisons (human, 4.0%; dog, 6.0%; mouse, 8.4%; rat, 4.4%; chicken, 1.8%; *T. nigroviridis*, 4.9%; zebrafish, 3.8%). In general, a greater number of *Ambystoma *orthologs were identified among amniote taxa versus fish taxa (Table 1). This suggests greater conservation of orthologs among tetrapod taxa.

To gain insight into variation in genome coverage of *Ambystoma*-vertebrate orthologies, we compared the distribution of *Ambystoma*-human orthologies to the expected distribution under random sampling of human loci (Table 2). We selected the human genome assembly for this comparison [25] because the assembly is relatively complete and contains a large number of gene annotations. The observed number of orthologs on three human chromosomes (HSA1, 12, and 17) deviated significantly from the expected number (p > 0.01). Notably, all three of these chromosomes contained an excess of orthologies, rather than a deficiency. A single human chromosome (HSA4) contained a marginally significant deficiency of *Ambystoma *orthologies (p = 0.50), however, given the large number of comparisons, a similar deviation would be expected to occur by chance. Comparisons with the human genome assembly suggest that *Ambystoma*-amniote orthologies will provide coverage of most regions of ancestral vertebrate genomes.

**Figure 2:**
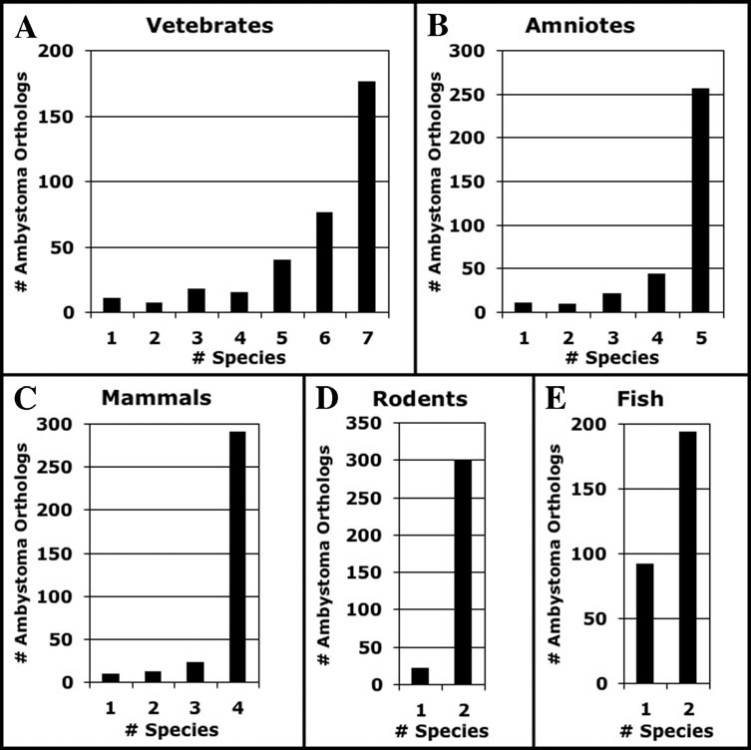
Frequency distributions of the numbers of presumptive *Ambystoma *orthologs that were identified among major vertebrate groups. Categories on the X-axes represent the total number of species within each major vertebrate group that yielded a presumptive *Ambystoma *ortholog.

Orthologs for a majority of *Ambystoma *marker sequences were identified in more than one reference genome (Figure 2). Of the 343 *Ambystoma *orthologs identified from all searches, 292 (85%) yielded hits to five or more genomes. A high proportion of *Ambystoma*-amniote orthologs were identified from four or more of the amniote taxa (88%). A lower proportion of *Ambystoma*-fish orthologs were identified from both fish taxa (68%). The relatively lower proportion of *Ambystoma-*fish orthologs may reflect lineage-specific gene losses and divergence that has occurred between these fish species, or differences in completeness of their genome assemblies. Below, we used *Ambystoma *orthologies as characters to identify conserved syntenies and gene orders and reconstruct key events in the evolution of vertebrate genomes.

### Conservation of synteny

The association index λ describes the extent to which chromosomal assignments of loci (genes) in one species are predictive of chromosome assignments in another species (see Methods). High λ values indicate high predictability; such values are expected when few inter-chromosomal rearrangements of genes occur between two species after divergence from a common ancestor. Thus, λ provides a measure of the combined effects of phylogenetic distance and lineage specific rearrangement rates on the inter-chromosomal distribution of genes. We estimated λ for pairwise comparisons between *Ambystoma *and each of the seven reference vertebrate genomes. Significant (non-zero) association indices were observed for all comparisons and there was considerable variation in λ values (0.18 for *Ambystoma *vs. zebrafish and mouse to 0.33 for *Ambystoma *vs. chicken; see Table 1). Variable λ values for *Ambystoma*-amniote comparisons illustrate the importance of lineage specific effects, because all amniotes share the same divergence time. In this case of λ variability among amniotes, lower λ values for *Ambystoma*-murid rodents indicate an increased rate of genome rearrangement in the murid rodent lineage.

**Figure 3:**
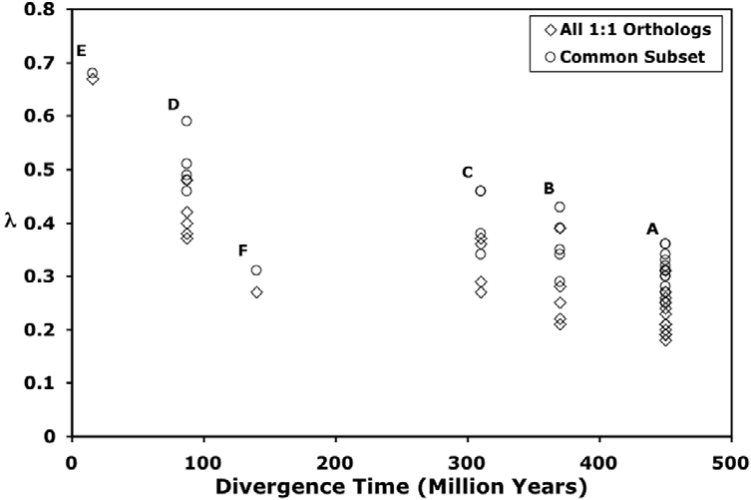
Plot of the λ association index versus the divergence time of vertebrate species. Labels A-F denote comparisons that hinge on the common ancestral nodes shown in Figure 1.

**Figure 4:**
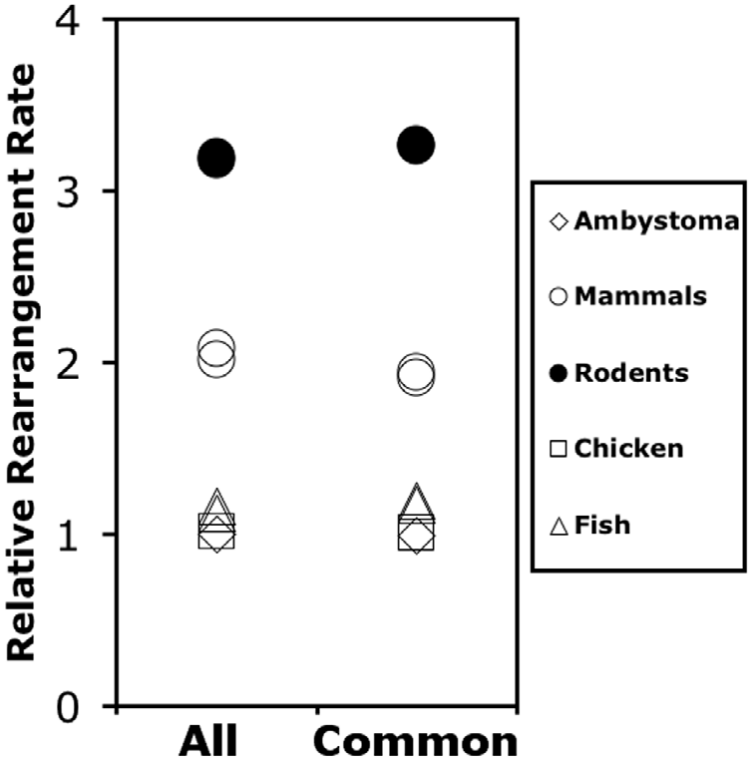
The average rate of decrease in λ for multiple vertebrate species, plotted relative to the *Ambystoma *rate. Rates are based on the set of all genes that showed 1:1 orthology in comparisons between *Ambystoma *each of the seven reference vertebrate genomes (All), and a smaller set of genes in which 1:1 orthology was established among all species (Common).

To obtain a more complete picture of genome similarity we calculated pairwise λ values for all possible species comparisons using two different datasets: 1) the set of all genes that showed 1:1 orthology in comparisons between *Ambystoma *each of the seven reference vertebrate genomes (ranging from N = 170 for chicken-zebrafish to N = 309 for *Ambystoma*-human), and 2) a smaller set of genes (N = 110) in which 1:1 orthology was established among all species. The cumulative gene set was expected to identify a greater number of associations while the smaller set controlled for comparison-wise differences among the gene sets used to estimate λ. The cumulative gene set yielded slightly lower values of λ than the smaller set. For both gene sets, λ was inversely correlated with phylogenetic distance (Figure 3) [see Additional file 2]. As before, we also observed that λ varied substantially among species with identical divergence times, consistent with lineage specific variation in rearrangement rates. Notably, λ values for the *Ambystoma*-chicken comparison are higher than or similar to λ values calculated between chicken and mammals, and similar to values calculated between murid rodents and non-rodent mammals, despite differences in divergence time among these comparisons on the order of 60–280 million years (Figure 1) [see Additional file 2]. To better understand the effect of divergence time on variation in λ, we estimated the average rate of decrease in λ [(1-λ)/divergence time)] for all pairwise comparisons. Figure 4 shows that interchromosomal rearrangement rates are strikingly higher in murids and more variable among mammals in comparison to all other vertebrate groups. In contrast, genome rearrangements in non-mammalian vertebrate species appear to accumulate at a similar, lower rate.

### Conservation of segmental homology

**Figure 5:**
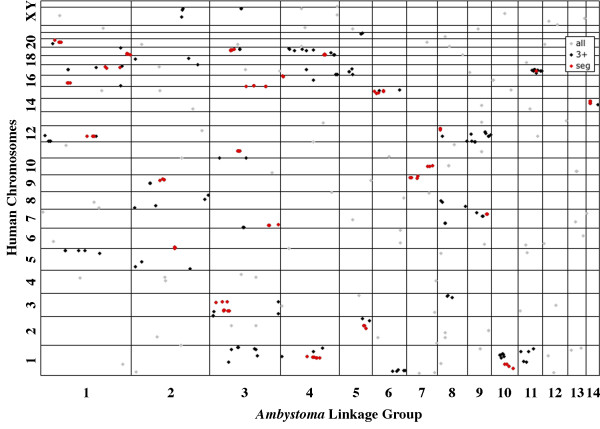
Oxford plot of the positions of presumptive orthologies between *Ambystoma *linkage groups and human chromosomes. Lines represent the boundaries of chromosomes (horizontal) and linkage groups (vertical). Dots represent the relative position of orthologs within the *Ambystoma *map. Orthologs that are members of synteny groups of three or more are shown in black. Orthologs that are members of significant (FISH) segmental homologies are shown in red. Some smaller chromosomes are not labeled. All chromosomes are presented ordinally from bottom to top.

**Figure 6:**
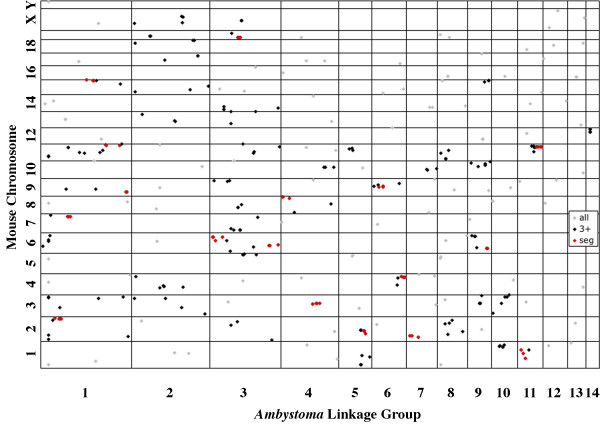
Oxford plot of the positions of presumptive orthologies between *Ambystoma *linkage groups and mouse chromosomes. See Figure 5 for further details.

**Figure 7:**
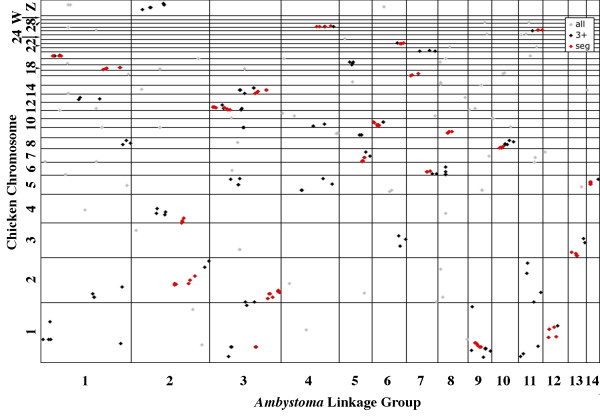
Oxford plot of the positions of presumptive orthologies between *Ambystoma *linkage groups and chicken chromosomes. The minichromosomes GGA16, 25, 29–31 and 33–38 are not shown; GGA16 contains no mapped *Ambystoma *orthologs and others are not represented in the genome assembly. See Figure 5 for further details.

**Figure 8:**
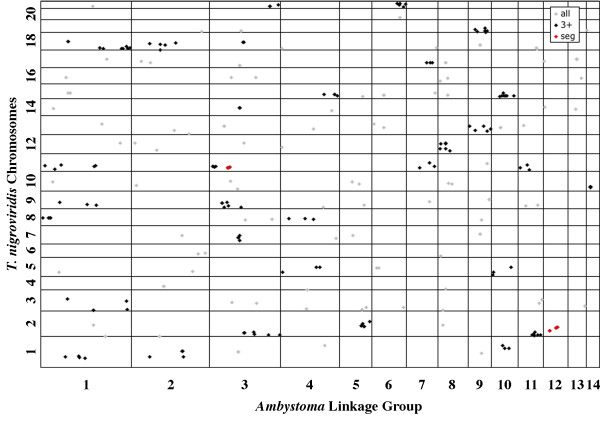
Oxford plot of the positions of presumptive orthologies between *Ambystoma *linkage groups and *Tetraodon nigroviridis *chromosomes. See Figure 5 for further details.

We compared the map position of *Ambystoma *genes to the physical positions of their presumptive orthologs in each of the seven reference genomes. These pair wise comparisons were visualized using oxford plots to show intra-chromosomal positions of orthologies between *Ambystoma *and each of the reference genomes (Figures 5, 6, 7, 8) [see Additional files 3, 4, 5]. In oxford plots, conserved segmental homologies can be identified as diagonally oriented clusters of points. We compared this visual approach with a statistical approach using the program FISH [26]. The algorithm underlying FISH appears to be somewhat conservative for *Ambystoma*-amniote comparisons because several clusters that are visually indicative of conserved segments were not identified as such, and several of the significant clusters did not always include orthologies that were very close to cluster margins. At any rate, the majority (57%) of the 334 *Ambystoma*-amniote orthologs were found within statistically significant, segmental homologies. The proportion of *Ambystoma *orthologs that were assigned to homologous segments varied greatly among comparative maps (Table 1) [see Additional file 1]. A much higher percentage of *Ambystoma*-amniote orthologs were found in significant segment homologies than *Ambystoma*-fish orthologs. For example, the *Ambystoma*-chicken oxford plot reveals a striking pattern of conservation of gene order (Figure 7). Overall, fewer segmental homologies were identified between *Ambystoma *and murid rodents vs nonrodent amniotes. However, the number of segmental homology differences among amniotes was small in comparison to the nearly 2-fold difference in λ values observed for *Ambystoma*-murids vs. *Ambystoma*-nonrodents (Table 1). Thus, although there has been greater reordering of loci among murid chromosomes during evolution, orders of loci within murid chromosomes are conserved and identifiable in comparisons to *Ambystoma*.

## Discussion

Amphibians occupy an important, intermediate position in the vertebrate evolutionary tree. Our study is the first to include amphibian gene order data in a taxonomically broad comparison of vertebrate genome structure. Comparisons of genome structure between *Ambystoma *and representative fish, reptilian, and mammalian species revealed extensive conservation of gene location at the intra- and inter-chromosomal levels. Overall, we identified conserved syntenies and segmental homologies for hundreds of *Ambystoma *protein-coding sequences [see Additional file 1]. These data provide evidence beyond nucleotide identity that *Ambystoma *genes are annotated with the correct vertebrate orthology. Information about gene orthology, conserved synteny, and segmental homology will extend *Ambystoma *as a research model because it will enable development of orthologous probes for comparative molecular studies, and the identification of candidate genes for *Ambystoma *mutants and QTL.

Our study shows that the *Ambystoma *Genetic Map can identify conserved syntenies and segmental homologies when compared to any of the primary vertebrate model organism genome assemblies. Overall, we found greater conservation of genome structure between *Ambystoma *and amniotes, however, many conserved syntenies are identifiable between *Ambystoma *and fish (*T. nigroviridis*, zebrafish). We also found that genome rearrangement rates are not simply a function of phylogenetic distance; there are clear differences in inter-chromosomal rearrangement rates, especially within mammals, as well as between mammals and "lower vertebrates". We elaborate on these points below and describe several new insights that amphibians provide concerning vertebrate genome evolution.

### Genome conservation between *Ambystoma *and fish

Fewer presumptive orthologs, conserved syntenies, and segmental homologies were identified between *Ambystoma *and fish (*T. nigroviridis*, zebrafish) than between *Ambystoma *and amniotes. This result is expected because of the deeper divergence time of *Ambystoma *and fish; in other words, there has been more time for nucleotide substitutions (that make it difficult to identify orthologs) and synteny disruptions to accumulate since the divergence of *Ambystoma *and fish from a common ancestor. Nevertheless, 57% of *Ambystoma *orthologs were observed in conserved syntenies with four or more orthologs in at least one fish species, and with the exception of *Ambystoma *linkage group (LG)13 (which shows strong synteny with GGA3), all *Ambystoma *linkage groups show discreet regions of synteny with chromosomes of *T. nigroviridis *and zebrafish. Assuming conservation of gene order during evolution, several regions of conserved synteny between *Ambystoma *and fish were likely present in the ancestral euteleostean genome. These include: the right hand portion of *Ambystoma *LG6, which shows extensive synteny with TNI21 and segmental homology with DRE19; and *Ambystoma *LG10, which shows extensive synteny with TNI15 and DRE20 (Figure 8) [see Additional file 5]. Observation of extensive synteny between *Ambystoma *and fish is interesting because recent evidence suggests a whole genome duplication predating the common ancestor of *T. nigroviridis *and zebrafish, followed by differential losses of paralogous loci [e.g. [7,27-29]]. Under such a model of genome evolution, the positions of syntenic *Ambystoma *genes are expected to map to overlapping positions on different fish chromosomes. We do observe this pattern for *Ambystoma*-*T. nigroviridis *orthologs on a few of the smaller *Ambystoma *linkage groups (e.g. *Ambystoma *LG9 vs. TNI13 and 19), however this pattern is not as obvious in larger *Ambystoma *linkage groups, or in comparisons between *Ambystoma *and zebrafish. The observed patterns appear to be consistent with chromosomal duplications in some instances, but may alternately reflect ancient large-scale rearrangements that have since been shuffled to yield interleaving sets of conserved syntenies. Better reconstruction of the pre-duplicated, ancestral teleost genome is needed to differentiate between these possibilities.

### Genome conservation between *Ambystoma *and amniotes

Results from our study indicate extensive conservation of gene orders between *Ambystoma *and amniotes, and especially between *Ambystoma *and chicken. Many of the orthologs identified on the smaller chicken chromosomes exist in nearly exclusive synteny or segmental homology with discreet regions of the *Ambystoma *genome (Figure 7). This is interesting because of the large difference in chromosome number and genome size between these species. *Ambystoma *has a much larger genome and haploid complement of 14 chromosomes [20], whereas chicken has a haploid complement of 39 chromosomes [9]. Because an ancestral chromosomal number of 12–14 chromosomes seems most likely for euteleost [7,28-31] tetrapod (Smith, unpublished data), and reptilian ancestors [32], differences between *Ambystoma *and chicken genomes are largely explained by lineage specific fissions (mostly giving rise to individual chicken microchromosomes) and a moderate number of large rearrangements. The very high number of segmental homologies observed between *Ambystoma*-chicken suggests they share a large portion of the ancestral tetrapod genome structure. When considering additional segmental homologies identified between *Ambystoma *and mammals, more than half of the *Ambystoma*-amniote orthologs that are currently located on the *Ambystoma *Genetic Map identify segmental homologies within at least one amniote genome, and by extension, the ancestral amniote and tetrapod genomes.

### Variation in interchromosomal rearrangement rates

Our study corroborates the idea that mammalian genomes are characterized by higher and more variable rates of genome rearrangement in comparison to other vertebrate groups [e.g. [31,33,34]]. In comparison to mammals, we estimated lower, but similar genome rearrangement rates for *Ambystoma*, chicken, zebrafish, and *T. nigroviridis*. Our estimates are consistent with cytogenetic data that indicate extensive conservation of the avian karyotype over approximately 80–100 million years of evolution [35-37], with estimates of genome rearrangement rates between chicken and mammals [34,38], and with comparisons between chicken and reptiles [38]. It is curious to find similar rearrangement rates among non-mammalian vertebrates that differ so greatly in life history and genome structure, and whose genomes have been shaped differently by lineage-specific processes during evolution. Birds, amphibians, and fish have very different generation times, chromosome numbers, and genome sizes. However, our results suggest relatively constant rates of genome rearrangement from the euteleost ancestor to the origin of mammals.

### Evolution of human chromosomes

In the remainder of the discussion we provide a few examples to show how *Ambystoma *provides perspective on the evolution of gene orders within the human genome. In general, *Ambystoma *comparative mapping data are useful because they help establish ancestral amniote and tetrapod gene orders. The *Ambystoma *ancestral perspective is needed to identify conserved syntenies and disruptions, and to corroborate evolutionary inferences based only on comparisons between chicken and mammals [9,33,40-43] or only mammals [33,44-46].

### Synteny of HSA1 and HSA19 loci in the ancestral amniote and tetrapod genomes

**Figure 9:**
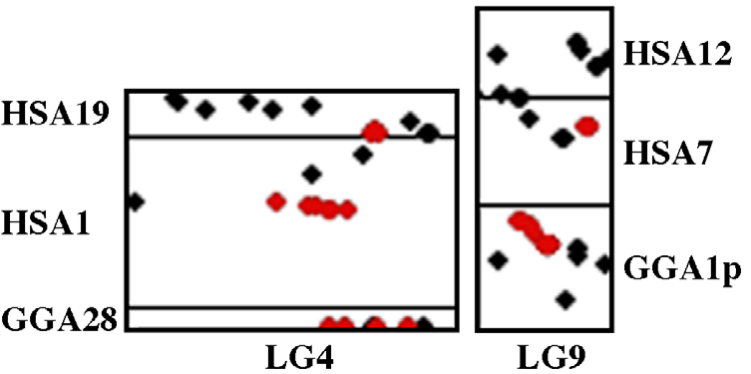
Oxford plot of the positions of presumptive orthologies between *Ambystoma *LG4 and 9, GGA1 and 28, and HSA1, 7, 12, and 19. The distribution of *Ambystoma*-chicken and *Ambystoma*-human conserved syntenies suggests that much of the content of *Ambystoma *LG4 and 9 was present in the ancestral tetrapod genome.

A region of segmental homology between *Ambystoma *LG4 and GGA28 overlaps regions of segmental homology between *Ambystoma *LG4 and two human chromosomes, HSA1 and HSA19 (Figure 9). This arrangement suggests that portions of HSA1 and 19 were joined in the ancestral tetrapod and amniote genomes. Fission of this ancestral gene order presumably occurred before the diversification of eutherian mammals (87 MYA) because *Ambystoma *LG4 orthologies are distributed similarly among the chromosomes of human, mouse, rat, and dog. The overall distribution of conserved syntenies among *Ambystoma *and amniotes indicates that many *Ambystoma *LG4 genes were syntenic in the ancestral tetrapod genome.

### Synteny of HSA7 and HSA12 loci in the ancestral amniote, tetrapod, and euteleost genomes

Regions of synteny and segmental homology between *Ambystoma *LG9 and GGA1 overlap the positions of syntenic markers located on HSA7 and 12 (Figure 9). This arrangement suggests that loci of HSA7 and 12 were syntenic in the ancestral tetrapod and amniote genomes. As was observed above for *Ambystoma *LG4, fission of this ancestral gene order presumably occurred before the diversification of eutherian mammals because *Ambystoma *LG9 orthologies are distributed similarly among the chromosomes of human, mouse, rat, and dog. Because *Ambystoma *LG9 also shows conserved synteny and segmental homology with much of DRE4, many *Ambystoma *LG9 genes were apparently syntenic in the euteleost ancestral genome.

### Value of multiple species in comparative genomics

*Ambystoma *LG12 and 13 show extensive conserved synteny and segmental homology with portions of GGA1 and 3, respectively. Apparently, these homologous chromosomal segments have changed little since diversification of the tetrapod lineage, approximately 370 million years ago. However, neither *Ambystoma *LG12 nor *Ambystoma *LG13 show substantial conserved synteny or segmental homology with any human chromosome. This suggests the possibility of lineage-specific synteny disruptions in the primate lineage, because *Ambystoma *LG12 does show conserved synteny with portions of the X-added region of rat and dog [47,48]. This example shows that conserved chromosomal segments may not always be identifiable in the human genome or other mammalian genomes; a multi-species perspective is essential to identify lineage specific effects in comparative vertebrate genomics.

### Fissions derived within the mammalian lineage

**Figure 10:**
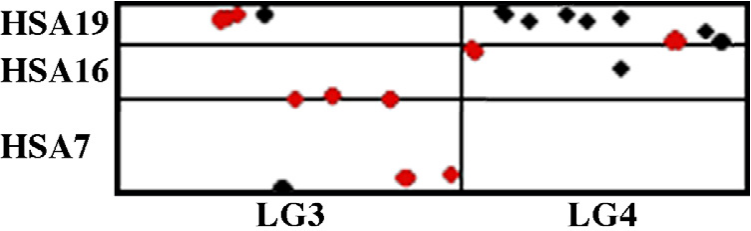
Oxford plot of the positions of presumptive orthologies between *Ambystoma *LG3 and 4 and HSA7, 16, and 19. The arrangement of *Ambystoma*-human orthologies suggests that some of the proposed ancestral chromosome arrangements of mammals can be traced back to the ancestral tetrapod genome.

Several pairs of human chromosomes may have been fused in the ancestral mammalian genome: HSA3/21, 4/8, 10/12, 7/16, 14/15, 16/19, and two regions of 12/22, [33,44-46]. Although our current dataset is insufficient to test all of these hypotheses, the *Ambystoma*-human oxford plot (Figure 5) suggests that two of these chromosome pairs were fused in the ancestral tetrapod and amniote genomes (Figure 10). Conserved syntenic regions of HSA16 and 19 adjoin each other in the middle of *Ambystoma *LG3 and at the left end of *Ambystoma *LG4. Additionally, conserved syntenic regions of HSA7 and HSA16 adjoin each other on the right end of *Ambystoma *LG3. Our data suggest that some of the chromosomal arrangements that have been proposed for the ancestral mammalian genome may trace back to the ancestral tetrapod genome.

## Conclusion

These studies demonstrate the importance of amphibians in revealing key events and trends in vertebrate genome evolution. Measurements of conserved synteny using *Ambystoma *orthologies suggest relatively constant rates of genome rearrangement from the euteleost ancestor to the origin of mammals. *Ambystoma *comparative mapping data are also useful in establishing ancestral amniote and tetrapod gene orders and identifying synteny disruptions that have occurred in amniote lineages. More than half of the *Ambystoma*-amniote orthologs that are currently located on the *Ambystoma *Genetic Map identify segmental homologies within at least one amniote genome, and by extension, the ancestral amniote and tetrapod genomes. Comparisons between *Ambystoma *and amniotes also reveal some of the key events that have structured the human genome since diversification of the ancestral amniote lineage.

## Methods

### Sequence comparisons

*Ambystoma *orthologs were identified from assembled contigs of the Salamander Genome Project [49-51] and other sequences published in GenBank [see Additional file 6]. These sequences ranged in length from 126 to 6167 bp and presumably correspond to partial and full-length RNA transcripts. A FASTA file of these sequences is included as a supplementary document [see Additional file 7]. Similarity searches and sequence alignments between translated *Ambystoma *sequences and translated genome sequences were performed using the program BLAT [52]. Alignments were generated between the source sequences for 491 *Ambystoma *genetic markers [21] and genome assemblies for human, mouse, rat, dog, chicken, zebrafish, and *T. nigroviridis*. Source sequences for human, mouse, rat, dog, chicken, zebrafish, and *T. nigroviridis *(respectively: hg17 build 35, mm6 build 34, rn3, canFam1, galGal2, danRer2, tetNig1 V7) were downloaded from the UCSC Genome Browser Gateway [53]. Cumulative bitscores were calculated for alignments between *Ambystoma *sequence and full genome sequences by summing across presumptive exons. This was accomplished by summing bitscores for otherwise continuous alignments that were interrupted by gaps of 10,000 or fewer bases.

### Statistical analysis of conserved synteny

Houseworth and Postlethwait [54] proposed two measures of synteny conservation: ρ and λ. Both of these statistics measure the degree of association between chromosomes (or other segments) from two genomes. The statistic ρ is equivalent to the square of Cramer's V statistic for frequencies of orthologs in a two-way table of chromosomes [54,55]. Cramer's V and ρ are scaled χ^2 ^statistics and as such may not be fully appropriate for measures of association when the average cell frequency within a two-way contingency table is less than 6 [56]. In other words accurate estimation of the χ^2 ^statistic for comparisons between two genomes with 1N = 20 would require at minimum identification of 2400 (20*20*6) orthologies. Furthermore, χ^2 ^based measures of association are not directly comparable between analyses, nor interpretable in a probabilistic sense [e.g. [57-60]].

In terms of pairwise comparisons between genomes, λ provides a measure of the proportional increase in ability to predict the chromosomal assignment of an ortholog in either of two species (or in probabilistic terms, "the relative decrease in probability of erroneous guessing"; [60], when the ortholog's position is known in the other species, *vs*. when it is unknown) [60]. The value of λ ranges from 0 to 1, with a value of λ = 0 representing the case where knowledge of the positions of orthologous loci in either species is completely uninformative in predicting the location of orthologs in the other, and a value of λ = 1 representing the case where knowledge of the positions of orthologous loci in either species can be used to exactly predict the location of all orthologs in the other. Values of ρ and λ were highly similar among our analyses. For simplicity and ease of interpretation, and because the λ statistic is seemingly more appropriate for the question at hand, we therefore report only values for λ with approximate 95% confidence estimated using the methods of Goodman and Kruskal [61].

### Statistical analysis of segmental homology

Segmental homologies were identified by comparing the positions of orthologs between the *Ambystoma *genetic map and the reference genomes for human, mouse, rat, dog, chicken, zebrafish, and *T. nigroviridis*. The *Ambystoma *map and reference genomes were formatted as concatenated (across linkage groups or chromosomes) series of orthologs and input into the program FISH [26]. In effect, FISH identifies segmental homologies by comparing the distribution of points on an oxford plot to the expected null distribution for an equal number of randomly scattered points. Concatenating chromosomes of multichromosomal genomes permits correct calculation of the null distribution of orthologies by FISH. However, one potential caveat of using concatenated genomes is that the analysis does not take into account the position of chromosomal boundaries. The possibility therefore exists that clusters or orthologies that cross the boundaries of chromosomes or linkage groups will be identified as segmental homologies. Because these putative clusters involve artificially generated segments, they likely represent spurious segmental homologies. To check for this possibility, the locations of all identified segmental homologies were examined manually. A single segmental homology in the *Ambystoma*-mouse comparison was observed that crossed a boundary. This homology was removed from subsequent analyses. We note that boundary-crossing clusters might alternately represent fission breakpoints that were placed (by chance) adjacent to one another in the concatenated genome. We intend to explore this possibility in future work.

## Abbreviations

LG – linkage group

## Authors' contributions

JJS and SRV conceived of the study and prepared the manuscript. JJS performed statistical analyses.

## Supplementary Material

Additional file 1 Summary statistics for *Ambystoma*-vertebrate genome alignments. This table provides alignment start and end positions and bitscore values for alignments between mapped *Ambystoma *sequences and genome assemblies for human, mouse, rat, dog, chicken, zebrafish, and *T. nigroviridis*. Click here for file

Additional file 2 Values of the λ association index for comparisons among all eight representative vertebrate species. This table provides values of the λ association index and 95% confidence intervals for all pairwise comparisons among *Ambystoma*, human, mouse, rat, dog, chicken, zebrafish, and *T. nigroviridis*. Click here for file

Additional file 3 Oxford plot of the positions of presumptive orthologies between *Ambystoma *linkage groups and rat chromosomes. This plot shows the relative position of orthologies in the *Ambystoma *(X-axis) and rat (Y-axis) genomes. See Figure 5 for further details. Click here for file

Additional file 4 Oxford plot of the positions of presumptive orthologies between *Ambystoma *linkage groups and dog chromosomes. This plot shows the relative position of orthologies in the *Ambystoma *(X-axis) and dog (Y-axis) genomes. See Figure 5 for further details. Click here for file

Additional file 5 Oxford plot of the positions of presumptive orthologies between *Ambystoma *linkage groups and zebrafish chromosomes. This plot shows the relative position of orthologies in the *Ambystoma *(X-axis) and zebrafish (Y-axis) genomes. See Figure 5 for further details. Click here for file

Additional file 6 FASTA identifiers, species, and GenBank GIs that are associated with *Ambystoma *markers. This table provides GenBank and species identifiers for all of the *Ambystoma *markers that were used in this study. Click here for file

Additional file 7 A FASTA file of *Ambystoma *sequence that were used in this study. This FASTA formatted file provides all of the *Ambystoma *sequences that were used to generate alignments in this study. Click here for file

## Figures and Tables

**Table 1:** Summary of sequence alignments and analyses of synteny and segmental homology using the full set of mapped *Ambystoma *sequences

Species	Divergence	Orthologs	BLAT	λ	% in
	Time^a^		Alignments		SH^b^
Human	370 MY	322	341	0.25 ± 0.12	32
Dog	370 MY	317	340	0.24 ± 0.12	25
Mouse	370 MY	308	350	0.18 ± 0.12	27
Rat	370 MY	316	340	0.19 ± 0.13	23
Chicken	370 MY	284	301	0.33 ± 0.13	34
*T.nigroviridis*	450 MY	243	255	0.23 ± 0.14	2
Zebrafish	450 MY	237	248	0.18 ± 0.15	3

Total Numbers		343	2175		57

^a^The approximate divergence time in millions of years (MY) between *Ambystoma *and the vertebrate species used in this study. See Figure 1 for references.

^b^SH = Segmental Homologies.

**Table 2:** Distribution of human/*Ambystoma *orthologies across human chromosomes

Human	Annotated	Human/Ambystoma Orthologies				
Chromosome	Genes^a^	Observed	Expected^b^	Deviation^c^	χ^2^	p
HSA1	2,580	47	33.3	+	5.67	0.017
HSA2	1,742	16	22.5	-	1.86	0.173
HSA3	1,378	21	17.8	+	0.59	0.443
HSA4	1,017	6	13.1	-	3.86	0.050
HSA5	1,188	10	15.3	-	1.85	0.174
HSA6	1,385	11	17.9	-	2.63	0.105
HSA7	1,367	21	17.6	+	0.65	0.421
HSA8	927	12	12	+	0.00	0.989
HSA9	1,069	12	13.8	-	0.23	0.631
HSA10	983	8	12.7	-	1.72	0.189
HSA11	1,692	15	21.8	-	2.13	0.145
HSA12	1,261	28	16.3	+	8.48	0.004
HSA13	491	3	6.3	-	1.75	0.186
HSA14	1,173	10	15.1	-	1.74	0.188
HSA15	884	11	11.4	-	0.01	0.906
HSA16	1,031	16	13.3	+	0.55	0.458
HSA17	1,354	29	17.5	+	7.63	0.006
HSA18	400	4	5.2	-	0.26	0.610
HSA19	1,584	28	20.4	+	2.81	0.094
HSA20	710	11	9.2	+	0.37	0.542
HSA21	337	4	4.3	-	0.03	0.869
HSA22	693	4	8.9	-	2.73	0.099
HSAX	1,125	13	14.5	-	0.16	0.693

^a^The number of genes that were assigned to each human chromosome (Build 35.1) [25].

^b^The expected number of human salamander orthologies is based on random sampling from the human genome. This number is calculated as the proportion of genes that occur on a given human chromosome multiplied by the entire number of human/*Ambystoma *orthologies that were identified (N = 340). A single HSAY orthology is excluded from this table.

^c^"+" designates a positive deviation from the expected number,"-" designates a negative deviation from the expected number.
