# Supplementary material for: Gene order data from a model amphibian (Ambystoma): new perspectives on vertebrate genome structure and evolution
Source: BMC Genomics. 2006 Aug 29;7:219. doi: 10.1186/1471-2164-7-219 (PMC1560138; doi:10.1186/1471-2164-7-219)
Supplement: Additional file 2 — Values of the λ association index for comparisons among all eight representative vertebrate species. This table provides values of the λ association index and 95% confidence intervals for all pairwise comparisons among Ambystoma, human, mouse, rat, dog, chicken, zebrafish, and T. nigroviridis. [file 1471-2164-7-219-S2.doc]

Supplementary Table 2 – Values of the λ association index for comparisons among all eight representative vertebrate species.

#### AM HS CF RN MM GG TN DR

AM - 0.28 ± 0.13 0.25 ± 0.13 0.21 ± 0.14 0.22 ± 0.13 0.39 ± 0.12 0.24 ± 0.15 0.18 ± 0.15

(309) (298) (302) (282) (245) (231) (228)

HS 0.35 ± 0.20 - 0.48 ± 0.10 0.40 ± 0.12 0.42 ± 0.11 0.37 ± 0.13 0.26 ± 0.14 0.19 ± 0.15

(278) (284) (267) (224) (214) (213)

CF 0.39 ± 0.20 0.59 ± 0.14 - 0.37 ± 0.11 0.38 ± 0.11 0.36 ± 0.13 0.27 ± 0.14 0.23 ± 0.14

(278) (264) (223) (213) (210)

RN 0.34 ± 0.20 0.49 ± 0.17 0.46 ± 0.17 - 0.67 ± 0.09 0.29 ± 0.14 0.20 ± 0.15 0.21 ± 0.15

(268) (220) (215) (214)

MM 0.29 ± 0.22 0.51 ± 0.17 0.48 ± 0.17 0.68 ± 0.14 - 0.27 ± 0.14 0.21 ± 0.15 0.19 ± 0.15

(212) (206) (200)

GG 0.43 ± 0.18 0.46 ± 0.17 0.46 ± 0.17 0.38 ± 0.19 0.34 ± 0.20 - 0.31 ± 0.15 0.25 ± 0.16

(176) (170)

TN 0.30 ± 0.21 0.32 ± 0.19 0.36 ± 0.18 0.31 ± 0.20 0.28 ± 0.21 0.36 ± 0.18 - 0.27 ± 0.15

(175)

DR 0.25 ± 0.21 0.31 ± 0.19 0.34 ± 0.20 0.30 ± 0.20 0.27 ± 0.20 0.33 ± 0.19 0.31 ± 0.20 -

____________________________________________________________________________________________________________

Values above the diagonal are based on the complete set of 1:1 *Ambystoma* orthologs that were identified in each species. The number of orthologs that were used for each comparison is provided in parentheses. Values below the diagonal are based on the set of 110 1:1 AM orthologs that were common to all species. Abbreviations for species names are: AM (*Ambystoma*), HS (human), CF (dog), RN (rat), MM (mouse), GG (chicken), TN (*T. nigroviridis*), DR (zebrafish).
