# Supplementary figures and images for: Gene order data from a model amphibian (Ambystoma): new perspectives on vertebrate genome structure and evolution
Source: BMC Genomics. 2006 Aug 29;7:219. doi: 10.1186/1471-2164-7-219 (PMC1560138; doi:10.1186/1471-2164-7-219)

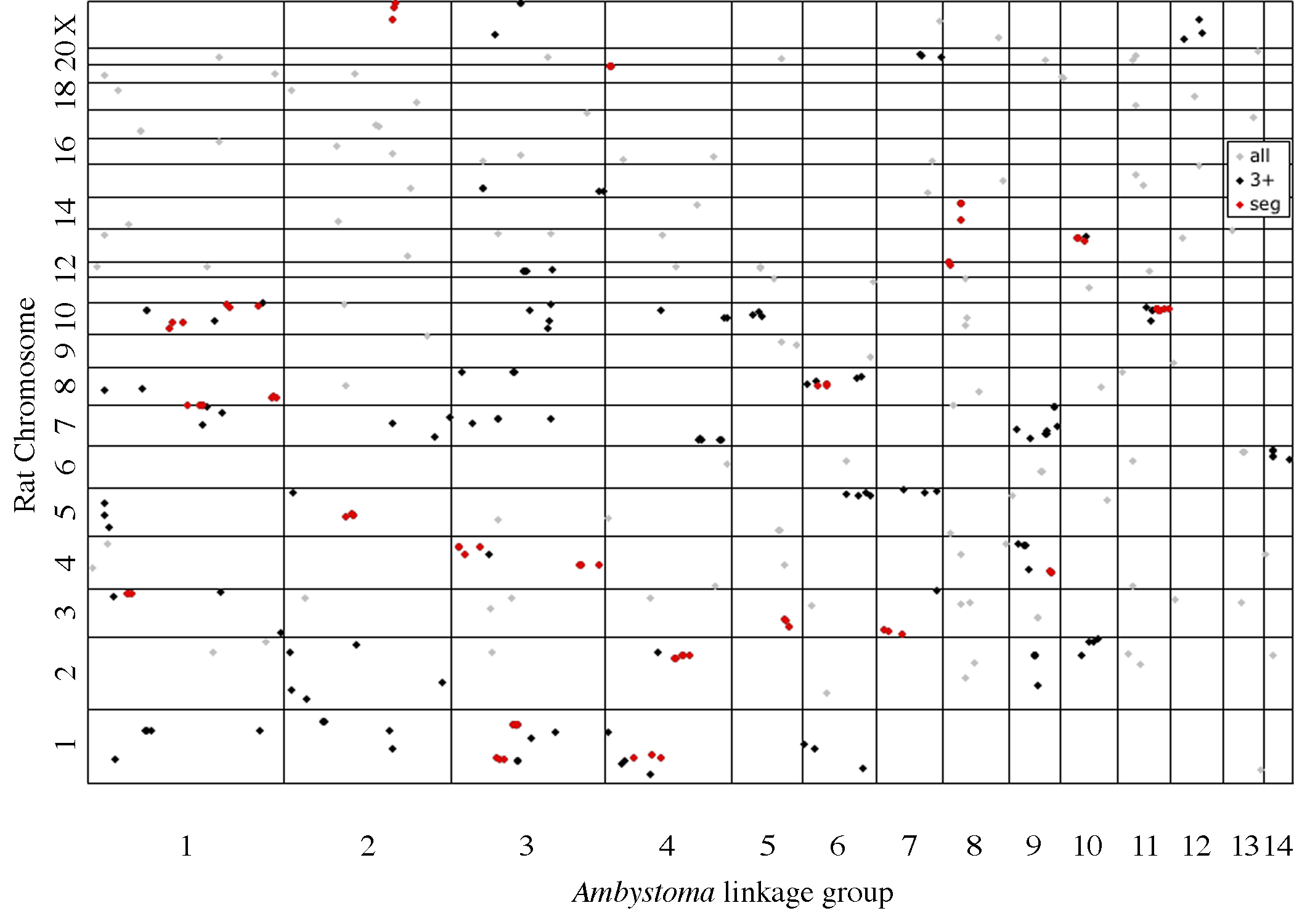

Supplement: Additional file 3 — Oxford plot of the positions of presumptive orthologies between Ambystoma linkage groups and rat chromosomes. This plot shows the relative position of orthologies in the Ambystoma (X-axis) and rat (Y-axis) genomes. See Figure 5 for further details. [file 1471-2164-7-219-S3.tiff]

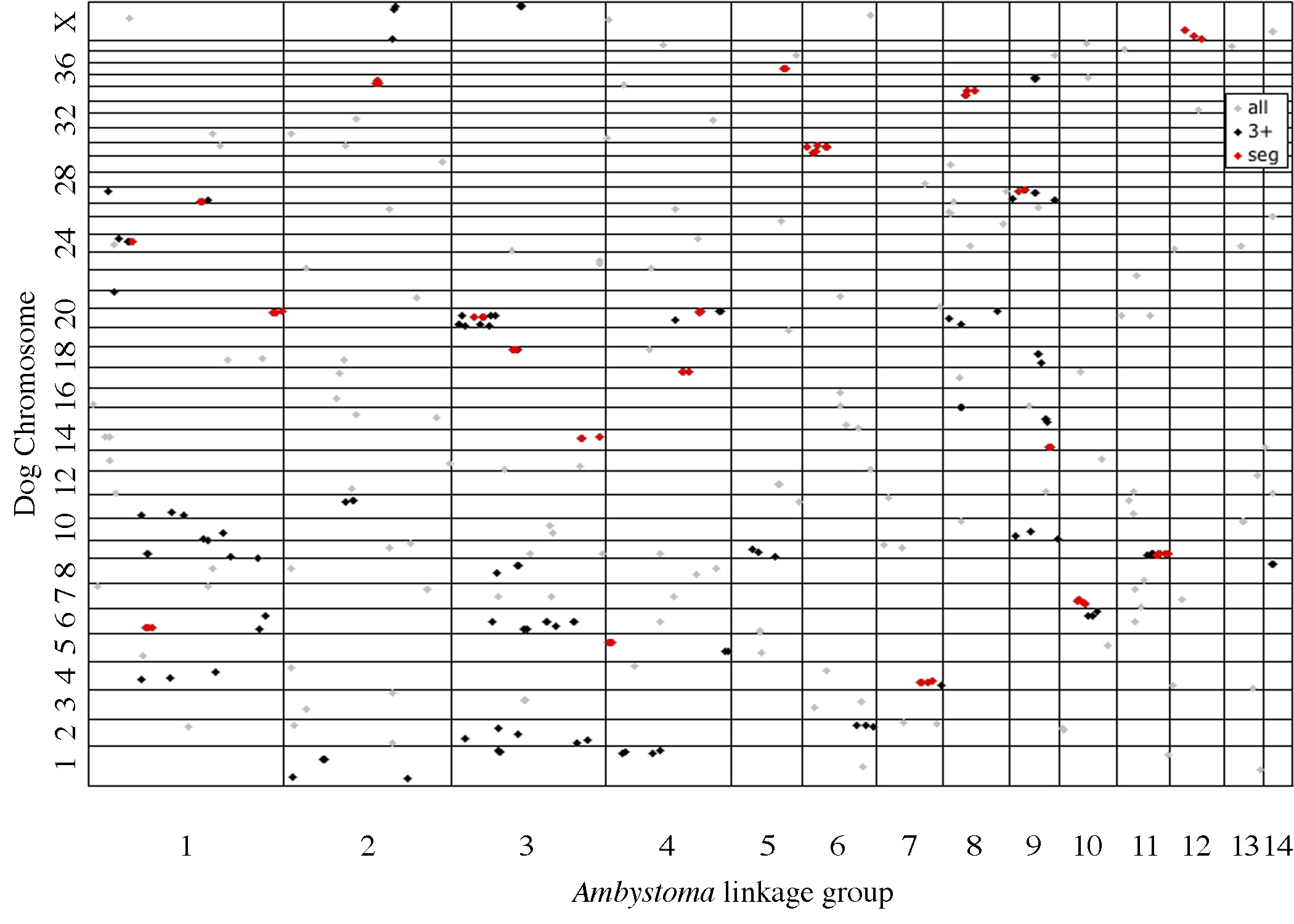

Supplement: Additional file 4 — Oxford plot of the positions of presumptive orthologies between Ambystoma linkage groups and dog chromosomes. This plot shows the relative position of orthologies in the Ambystoma (X-axis) and dog (Y-axis) genomes. See Figure 5 for further details. [file 1471-2164-7-219-S4.tiff]

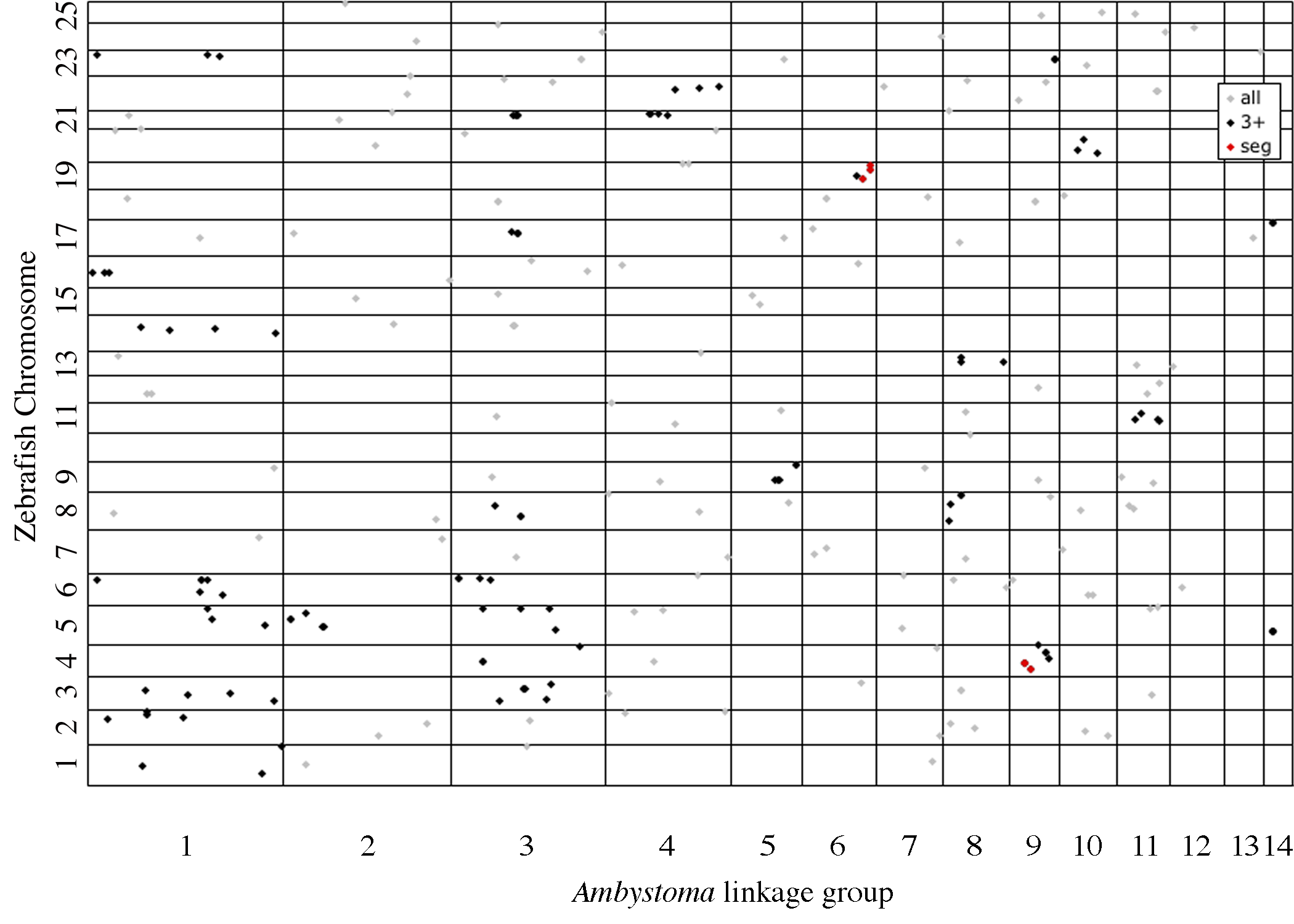

Supplement: Additional file 5 — Oxford plot of the positions of presumptive orthologies between Ambystoma linkage groups and zebrafish chromosomes. This plot shows the relative position of orthologies in the Ambystoma (X-axis) and zebrafish (Y-axis) genomes. See Figure 5 for further details. [file 1471-2164-7-219-S5.tiff]
